# Homozygosity mapping reveals novel and known mutations in Pakistani families with inherited retinal dystrophies

**DOI:** 10.1038/srep09965

**Published:** 2015-05-06

**Authors:** Muhammad Arif Nadeem Saqib, Konstantinos Nikopoulos, Ehsan Ullah, Falak Sher Khan, Jamila Iqbal, Rabia Bibi, Afeefa Jarral, Sundus Sajid, Koji M. Nishiguchi, Giulia Venturini, Muhammad Ansar, Carlo Rivolta

**Affiliations:** 1 Department of Biochemistry, Faculty of Biological Sciences, Quaid-i-Azam University, Islamabad 45320, Pakistan; 2 Department of Medical Genetics, University of Lausanne, 1005 Lausanne, Switzerland; 3 Department of Ophthalmology, Tohoku University Graduate School of Medicine, Sendai, Miyagi 980-8574, Japan; 4 Pakistan Medical Research Council, Islamabad, 44000, Pakistan

## Abstract

Inherited retinal dystrophies are phenotypically and genetically heterogeneous. This extensive heterogeneity poses a challenge when performing molecular diagnosis of patients, especially in developing countries. In this study, we applied homozygosity mapping as a tool to reduce the complexity given by genetic heterogeneity and identify disease-causing variants in consanguineous Pakistani pedigrees. DNA samples from eight families with autosomal recessive retinal dystrophies were subjected to genome wide homozygosity mapping (seven by SNP arrays and one by STR markers) and genes comprised within the detected homozygous regions were analyzed by Sanger sequencing. All families displayed consistent autozygous genomic regions. Sequence analysis of candidate genes identified four previously-reported mutations in *CNGB3, CNGA3, RHO*, and *PDE6A*, as well as three novel mutations: c.2656C > T (p.L886F) in *RPGRIP1*, c.991G > C (p.G331R) in *CNGA3,* and c.413-1G > A (IVS6*-*1G > A) in *CNGB1*. This latter mutation impacted pre-mRNA splicing of *CNGB1* by creating a -1 frameshift leading to a premature termination codon. In addition to better delineating the genetic landscape of inherited retinal dystrophies in Pakistan, our data confirm that combining homozygosity mapping and candidate gene sequencing is a powerful approach for mutation identification in populations where consanguineous unions are common.

Inherited retinal dystrophies (IRDs) are a group of rare genetic disorders for which mutations in causative genes result either in the degeneration or the dysfunction of retinal cells. In the majority of cases, they are progressive conditions that can lead to legal or complete blindness^1^. IRD phenotypes are rather heterogeneous in terms of onset, progression, and severity of the disease. Symptoms and signs may be mild and stationary, such as for example in congenital stationary night blindness and achromatopsia, or progressive and severe, such as in retinitis pigmentosa (RP) and cone and cone-rod dystrophies^2^. Genetically, IRDs are also heterogeneous, with more than 190 responsible genes reported to date^3^. Furthermore, IRDs are inherited as an autosomal recessive, autosomal dominant, or X-linked trait, with autosomal recessive being the most prominent one^3^.

Homozygosity mapping is an efficient tool to map regions harboring either novel or known recessive mutations^4^ and is particularly effective in consanguineous families or in populations that are geographically isolated and are prone to result in a high rate of endogamy^5^. Although homozygosity mapping is not a recent technique, introduction of SNP-based genotyping microarrays has provided a fast and effective means of analysis, as demonstrated by a number of studies already^5^^,^^6^^,^^7^^,^^8^. It is also particularly effective for detecting IRDs mutations because of the rather elevated frequency of heterozygous recessive IRD variants in the general population^9^.

IRD diagnosis and genotype-phenotype correlations can be extremely daunting tasks, especially in developing countries. This study was designed to apply homozygosity mapping in consanguineous Pakistani families segregating IRDs that were minimally characterized from a clinical standpoint with the aims of (i) identifying the causative genetic agents of the disease and (ii) helping clinical diagnosis of patients.

## Materials and Methods

### Ethics statement

This study was designed in compliance with the tenets of the Declaration of Helsinki and carried out according to protocols that were approved by the Institutional Review Boards of Quaid-i-Azam University, University of Lausanne, and Tohoku University. Written informed consent for providing medical information and blood samples was obtained from each participant.

### Families and preparation of samples

**Figure 1:**
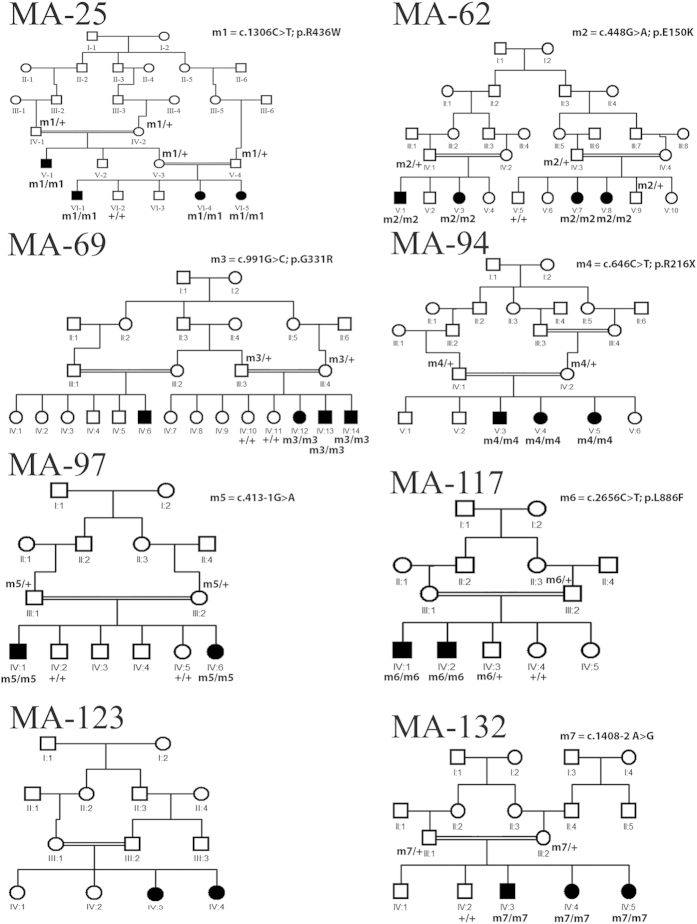
**Overview of the pedigree structure of the Pakistani families participating in this study.** Affected individuals are indicated with filled symbols, whereas unaffected relatives are indicated by open symbols. +, wild type allele; m#, mutation.

Families with two or more affected individuals were ascertained by physicians and scientists from the Quaid-i-Azam University, who visited them at their places of residence. Out of eight pedigrees, six (MA25, MA69, MA94, MA117, MA123 and MA132) were enrolled from rural areas of the Punjab province, while two (MA62 and MA97) were from the Sindh province. Pedigrees were drawn (Fig. 1) and a standard questionnaire was used to collect information including: family history, visual complaints, pattern of disease inheritance, and assessment of additional non-ocular clinical signs such as polydactyly, male infertility, renal and hearing impairment. Further fundoscopic examination and electroretinography (ERG) was performed when available.

Blood samples of affected and unaffected individuals from each family were collected on site. DNA purification was carried out using standard organic phenol-chloroform extraction methods. For low-volume samples, the NucleoSpin blood extraction kit (Macherey-Nagel, Bethlehem, PA) was used.

### Homozygosity mapping

Seven families (MA62, MA69, MA94, MA97, MA117, MA123 and MA132) were genotyped by using the Illumina HumanCytoSNP-12v2.1 SNPs array (Illumina, Santa Clara, CA, USA), containing ~300,000 markers, at the NCCR Genomics Platform of the University of Geneva, Switzerland. Arrays were processed according to manufacturer’s protocols. The SNP data were analyzed by using HomozygosityMapper^10^ and homozygous regions shared by affected individuals of each family were further assessed to explore involvement of genes known to be implicated in IRDs’ molecular pathology. An additional family, MA25, was genotyped by using highly polymorphic microsatellite markers encompassing known achromatopsia candidate genes, as described previously^11^.

### Molecular analysis

The entire open reading frame (ORF) and exon-intron boundaries of candidate genes were screened by means of PCR-amplification and Sanger sequencing in probands of each family. Primers (Supplementary Table S1) were designed by using the Primer 3 software^12^ and PCR amplification was performed under standard conditions, with an annealing step at 57 °C for 30 seconds that was common to all primer pairs. PCR products were purified by treatment with the ExoSAP-it reagent (Affymetrix, Santa Clara, CA) and sequenced using the Big Dye Terminator Cycling Sequencing Kit v3.1 (Applied Biosystem, Foster City, CA) by an ABI 3130xl Genetic Analyzer (Applied Biosystems). Sequencing data were analyzed using the CLC Bio software (Qiagen, Boston, MA) and compared with the corresponding human reference sequence (build hg19). Co-segregation analysis of all mutations was done in all families. Novel DNA variations were compared with data from public databases (Exome Variant Server, EVS^13^ and 1000 genomes^14^) and with information obtained by sequencing 200 healthy controls from different ethnic groups from all provinces of Pakistan. In order to evaluate the putative pathological nature of the novel missense variants reported in this study, we used three in silico tools, namely Polymorphism Phenotyping v2 (Polyphen-2)^15^, Sorting Intolerant from Tolerant (SIFT)^16^, and Mutation Taster^17^.

RPGRIP1 protein sequences from different species including human (*H. sapiens*, NP_065099.3) macaque (*M. mulatta*, XP_002808500.1) mouse (*M. musculus*, NP_076368.1), cow (*B. taurus*, NP_851377.1), dog (*C. lupus familiaris*, XP_851597.2), Xenopus (*X. tropicalis*, XP_002933948.2) and zebrafish (*D. rerio,* ENSDARP00000118806) were aligned using the CLC Genomics Workbench (Qiagen) in order to check the evolutionary conservation of the substituted amino acid in RPGRIP1. The same procedure was applied to CNGA3 protein sequences (human, NP_001289.1; macaque, XP_001101944.2; mouse, NP_001268939.1; cow, NP_776704.1; dog, XP_538462.3; Xenopus, XP_002931690.2; zebrafish, XP_005166141.1).

### Splicing variant analysis

To predict the putative impact of the identified splice site variation c.413-1G > A in *CNGB1*, in silico analysis was done using MutPred Splice (v1.3.2)^18^, Human Splice Finder (v2.4.1)^19^ and SKIPPY^20^. In silico results were experimentally validated by means of a minigene assay. More specifically, a genomic DNA region spanning introns 4 to 8 of *CNGB1* from patient MA97/IV-1 and one healthy control individual was PCR-amplified using the High Fidelity Phusion polymerase (Thermo Scientific, Pittsburgh, PA) for which a distinct primer pair (forward: 5′-AAGGTACCGGGGAGACAGTGGTTTAGGA-3′ and reverse: 5′-AATCTAGAACAGTCACTCCTCCCCATAGA-3′) was designed. The resulting PCR-products were subsequently cloned into the pcDNA 3.1/V5-His TOPO vector using the TOPO TA Cloning according to manufacturer’s protocol (Life Technologies, Carlsbad, CA). Plasmids were analyzed by direct Sanger sequencing and then transfected into HeLa cells. Total RNA was extracted using the Nucleospin RNA extraction kit (Macherey-Nagel), retrotranscribed with the Superscript III reverse transcriptase (Invitrogen, Carlsbad, CA), and the resulting cDNA was PCR-amplified by using primers lying within exon 5 (forward: 5′-AGGGTACTGACCTGGCTCAT-3′) and exon 8 (reverse: 5′-CAGATTCTGCTCCAGCCACA-3′) of the gene. The amplified products were separated by electrophoresis on a 2% agarose gel and were subsequently analyzed by Sanger sequencing.

## Results

### Clinical examination

**Figure 2:**
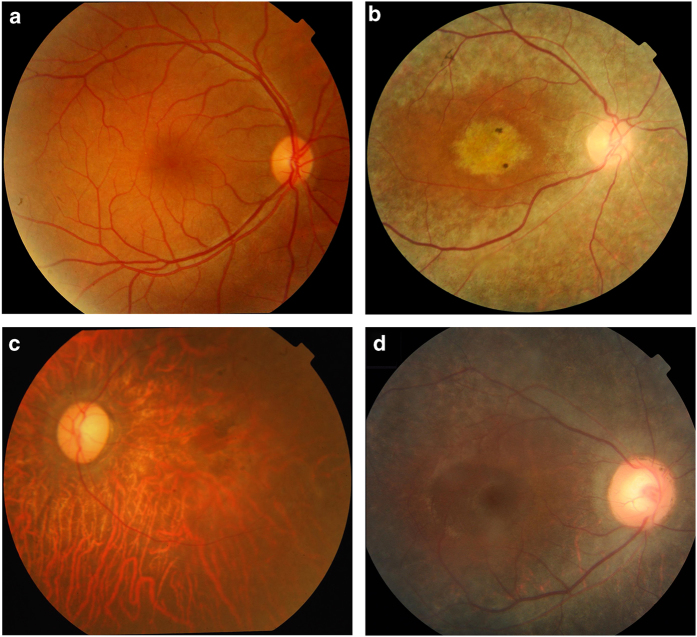
**Fundus photographs of affected individuals from families MA69, MA117, MA97 and MA132.** (**a)** Right eye of affected individual IV-12 of family MA69 (age 15 years) shows essentially a normal fundus except for the loss of foveal reflex. (**b)** Right eye of affected individual IV-2 of family MA-117 (age 47 years). Diffuse pigmentary retinal degeneration, attenuated vessels, and optic disc pallor accompanied by macular degeneration are present. (**c)** Left eye of affected individual IV-1 of family MA97 (age 24 years). Diffuse atrophic changes of the retinal pigment epithelium with pigment deposits and vascular attenuation are seen. (**d)** Right eye of affected individual IV-3 of family MA132 (age 18 years) showing diffuse pigmentary retinal degeneration sparing the macula, narrowed vessels, and optic disc pallor.

Due to the diversity of geographical origins of patients and the scarcity of diagnostic means available in rural Pakistan, the extent of clinical examination varied greatly across individuals (Table 1). The clinical presentation of patients in families MA25, MA69, and MA94 was consistent with that of achromatopsia, i.e. early onset of symptoms (<6 months of age) including photophobia, nystagmus and a complete absence of color discrimination. Fundus examination of individual IV-12 (aged 15 years) of MA69 showed essentially a normal fundus except for the loss of foveal reflex, indicating the presence of a modest maculopathy or a foveal hypoplasia (Fig. 2a). In MA117 and MA123, affected members had also photophobia and color vision loss, but the onset of their symptoms was reported during the second decade of life, suggestive of cone or cone-rod dystrophy. Patient IV-2 (aged 47 years) of MA117 also had fundus examination, showing macular degeneration as well as a widespread retinal degeneration accompanied by vascular attenuation and waxy pallor of the optic nerve head (Fig. 2b). None of the affected individuals from these families experienced night blindness.

In contrast, the clinical picture of families MA62, MA97 and MA132 showed the presence of RP-like symptoms, for which affected individuals initially experienced night blindness with eventual progressive visual loss. Specifically, affected members of family MA97 reported significant vision loss between 15 to 17 years of age, resulting in the end in legal blindness. Funduscopy of patient IV-1 (24 years old) revealed retinal degeneration with diffuse atrophy of the retinal pigment epithelium with macular involvement, scattered retinal pigment depositions vascular attenuation, and a modest pallor of the optic nerve head (Fig. 2c). This patient has self-reported myopia. The fundus appearance of patient IV-3 from family MA132 (18 years old) showed attenuated vessels, modest disc pallor, and diffuse atrophy of the retina and the retinal pigment epithelium with occasional pigment deposits sparing the macular area (Fig. 2d).

### Homozygosity mapping and mutation analysis

**Figure 3:**
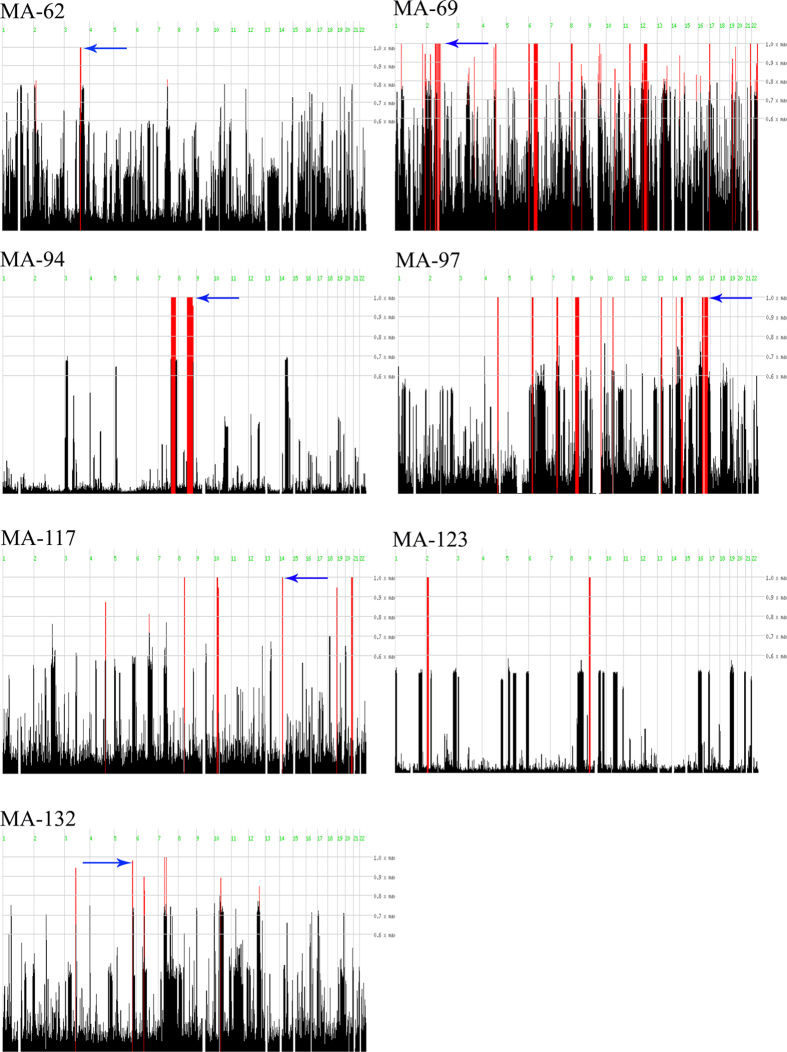
**Overview of homozygosity mapping results.** Data are shown for the seven families that were genotyped via SNP arrays and analyzed with HomozygosityMapper. The red lines indicate homozygous regions shared by affected individuals in each family. The arrows indicate the homozygous regions harboring genes in which pathogenic variants were identified.

DNA samples of all available affected and healthy members of the families studied were subject to whole genome SNP genotyping and homozygosity mapping (Supplementary Table S2), except for family MA25 (see below). Our analysis revealed several large homozygous regions that were shared among the affected members within the same family (Supplementary Table S3). In particular, regions containing more than 300 consecutive homozygous SNPs, on average corresponding to a genomic size of 1 Mb or larger, were prioritized. Family MA62 had a single homozygous peak, while others displayed two (MA94 and MA123) or multiple peaks (MA69, MA97, MA117 and MA132) (Fig. 3). In families where more than one peak of homozygous regions were observed, regions encompassing known IRD genes were selected for direct Sanger sequencing.

All of the DNA variants identified by sequencing, described below, were identified in homozygous state and co-segregated perfectly with affected individuals within the respective families, as expected. Details are reported in Table 2.

In family MA62, a single peak on chromosome 3 that comprised the Rhodopsin gene (*RHO/*NM_000539) was detected, and sequencing revealed a known missense mutation c.448G > A (p.E150K)^21^. Similarly, in family MA94 there were two homozygous stretches on chromosomes 7 and 8, spanning the genomic regions where known IRD genes *IMPDH1* and *CNGB3*, respectively, are located. Sequencing of the coding region of *CNGB3* (NM_019098) showed the presence of the previously-reported mutation c.646C > T (p.R216X)^22^. In MA132, a homozygous region on chromosome 5 contained the known RP gene *PDE6A* (NM_000440) and sequencing revealed the presence of the described mutation c.1408-2A > G (IVS10-2A > G)^23^. In MA123, one of the two detected homozygous peaks (on chromosome 16) comprised *KCNV2*, which is associated with cone-rod dystrophy^24^. However, screening of the ORF and exon-intron boundaries failed to identify any putative pathogenic variants, indicating that the culprit for IRD in this family is either a novel disease gene or a mutation in *KCNV2* that was not detectable by the methodology used in our analysis (e.g. a large structural variation, a mutation in deep intronic sequences, etc.).

Homozygosity mapping of family MA25 was done using highly polymorphic microsatellite markers spanning known achromatopsia loci. Analyses revealed a homozygous region between microsatellites D2S2333 and D2S1343, containing *CNGA3* (NM_001298). Sanger sequencing of the gene’s ORF revealed the known missense mutation c.1306C > T (p.R436W) in exon 8^25^,^26^.

**Figure 4:**
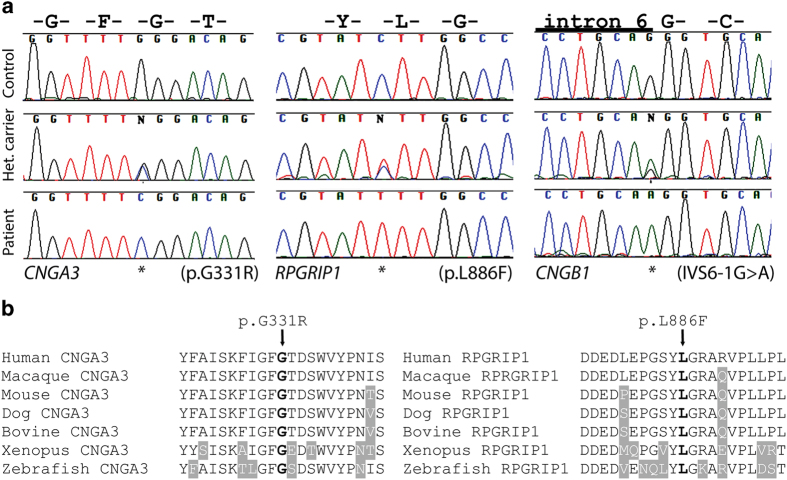
**Electropherograms of novel IRD mutations and amino acid sequence alignment of parts of human *CNGA3* and RPGRIP1.** (**a**) DNA sequences from control individuals, unaffected heterozygous carriers, and patients are shown. Mutated nucleotides are indicated by asterisks. The *CNGA3* mutation was detected in family MA69, the *RPGRP1* mutation in family MA117, and the *CNGB1* mutation was found in family MA97. (**b**) Human sequences are aligned with orthologous proteins from other vertebrates. Ten upstream and 10 downstream amino acids of the respective missense variants p.G331R and p.L886F are depicted. Residues identical to the human sequence across all sequences are black on a white background whereas different amino acids are indicated in white on a grey background. The amino acid residue at the position of the missense change is indicated in bold.

In MA69, multiple peaks were observed, but a 18.7 Mb region on chromosome 2 contained *CNGA3*. Sanger sequencing identified a novel homozygous missense change, c.991G > C (p.G331R) (Fig. 4). Similarly, in family MA117, a homozygous stretch on chromosome 14 between markers rs7148898 to rs12892350 harboring the known IRD gene *RPGRIP1* (NM_020366), was identified. Sequencing of *RPGRIP1* revealed a novel missense variation c.2656C > T (p.L886F) in exon 17 (Fig. 4). Neither c.991G > C in *CNGA3* nor c.2656C > T in *RPGRIP1* were present in any public databases, including the EVS and the 1000 genomes project. Both changes affect fully conserved amino acids from human to fish (Fig. 4). Furthermore, all in silico tools for the prediction of missense variants pathogenicity, namely Polyphen, SIFT, and Mutation Taster, predicted the changes to be probably damaging, deleterious, and disease-causing, respectively.

**Figure 5:**
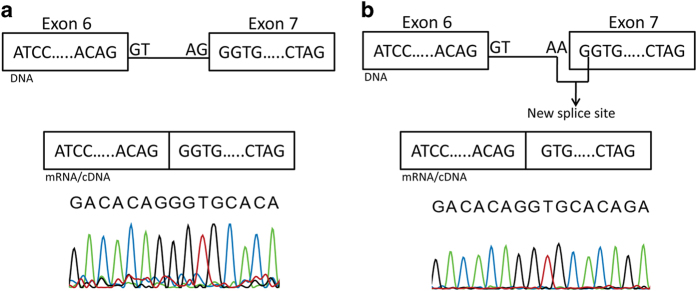
**Schematic representation of the effect of the splice acceptor site mutation c.413-1G > A on CNGB1 messenger RNA.** cDNA sequence from a construct obtained from control DNA is indicated in panel **a**, whereas cDNA from a construct bearing the IVS6-1G > A mutation is shown in panel **b**.

For family MA97, our attention was caught by a homozygous peak on chromosome 13 that contained *CNGB1*, a gene linked with RP. Sequencing revealed the splice site variation c.413-1G > A (IVS6-1G > A) affecting the invariant acceptor site of intron 6, which was never previously linked to disease (Fig. 4). Interestingly, this nucleotide change was present in dbSNP (rs189234741) but was observed only once in 5,000 chromosomes. Furthermore, it was detected in the framework of the 1,000 Genomes project, genotyping phase 1 (low coverage sequencing), indicating a possible technical artifact. Indeed, in silico analyses clearly confirmed that this variant should have a strong impact on the normal splicing pattern of the gene, as expected for DNA changes involving the -1 and -2 bases of intron acceptor sequences (scores: 0.394, 0.74, and “site broken” by Skippy, MutPred Splice, and Human Splice Finder, respectively). Transfection of HeLa cells with minigene constructs bearing this change and its wild-type counterpart revealed that IVS6-1G > A did affect the canonical splicing of exon 7 by knocking down its natural 5’ splice site and eliciting the use of a cryptic splice site, just one base pair downstream of intron 6’s acceptor sequence (Fig. 5). This event led to the loss of the first nucleotide of exon 7, producing a -1 frameshift and a premature stop codon 413 bases downstream of it (p.C139AfsX138/NP_001288.3).

Further screening of probands from 50 additional Pakistani pedigrees with IRD for all these mutations failed to reveal any additional positive subject (not shown).

## Discussion

Despite recent technological advances, molecular diagnosis of IRDs remains a challenging task, because of their high genetic and phenotypic heterogeneity. This is particularly true for laboratories that have no access to next-generation sequencing platforms and large-scale screening systems, and therefore have to assess candidate IRD genes one by one. Luckily, geographic isolation, consanguinity, or endogamy may increase the prevalence of particular mutations in selected populations, which can be pinpointed by homozygosity mapping. The rationale for this approach is that unaffected parents who have some degree of relatedness, are from a geographical isolate, or belong to an ethnic group for which endogamy is frequent, could be heterozygotes for the same recessive mutation from a common ancestor. This mutation, which at the population level may even be very rare, can be brought to homozygosity because of consanguinity and cause disease in these parents’ offspring. Since meiotic recombination affects chromosomes at a megabase scale, the mutation is co-inherited with large surrounding haplotypic blocks that are homozygous in patients and easily recognizable via the analysis of SNP alleles.

In this study we take advantage of the power of homozygosity mapping to identify mutations in Pakistani families with IRDs, originating from different geographical regions of the country and displaying consanguinity. In agreement with other studies^27^^,^^28^^,^^29^ our results indicate that indeed genomic regions harboring IRD genes can be efficiently highlighted by this technique and that very few candidate sequences have to be screened to reach molecular diagnosis. Consanguinity, however, is a key factor. Other cohort studies in consanguineous populations (e.g. Saudi Arabia), have shown that mutations in known retinal dystrophy genes were identified in >75% of the patient tested^30^,^31^. In outbred populations, where consanguinity is not common (e.g. the Netherlands), this success rate was considerably lower (in the range of 10–15%)^32^. Nonetheless, it should be noted that autozygome-guided mutation analysis has some limitations, since only homoallelic mutations are identified by this method. Thus, compound heterozygosity for recessive mutations, heterozygosity for dominant mutations, as well as hemizygosity for X-linked mutations are generally missed.

Concerning the variants identified, the change c.991G > C (p.G331R) in *CNGA3* detected in family MA69 affects a highly conserved glycine residue and, as the majority of previously-identified mutations, is a missense^33^. *CNGA3* encodes a channel protein which consists of six transmembrane helices, a pore region, a C-linker, and cyclic nucleotide-binding domain^34^. It is highly conserved in different species and mutations in this gene have been linked to achromatopsia^35^ a disease that causes symptoms that are compatible with those described in members of this family. The facts that (i) family MA69 displayed homozygosity for the *CNGA3* region, (ii) the DNA change identified involved a conserved amino acid residue and was absent in healthy controls and, despite we could not perform a detailed clinical examination, (iii) symptoms of patients corresponded to those of achromatopsia, which (iv) is an extremely rare disease, all strongly suggest that p.G331R is a pathogenic mutation.

The same arguments can be made for *RPGRIP1* c.2656C > T (p.L886F), detected in family MA117. RPGRIP1 is a ciliary protein composed of three different regions: an N-terminal coiled-coil domain, a central part containing two protein kinase C conserved region 2 (C2) motif, and a C-terminal region having a RPGR interacting domain^36^. The p.L886F missense change is located in the second C2 domain of RPGRIP1, which also harbors the majority of previously reported missense mutations^36^. Signs and symptoms of affected members are also compatible with those of cone-rod dystrophy, which can indeed be caused by *RPGRP1* mutations.

The variant c.413-1G > A (IVS6-1A > G) of *CNGB1* identified in family MA97 was predicted in silico and verified in vitro to alter the splicing pattern of this gene, inducing the use of a cryptic splice site and finally producing a homozygous frameshift that leads to a premature termination codon. Since this acquired stop codon occurs before the last exon, mutant mRNA likely undergoes nonsense-mediated decay and therefore produces no or very little protein^37^. Once again, the clinical picture of the affected members of family MA97 is compatible with RP and is similar to that of patients with previously-identified *CNGB1* mutations^38^,^39^.

Two families (MA62 and MA132) with mutations in *RHO* and *PDE6A*, respectively, also showed typical symptoms of RP. The c.448G > A/p.E150K mutation in *RHO* identified in MA62 was initially found in an Indian family^21^ but later Azam and colleagues also reported it in two separate Pakistani families^40^. Genotype analysis of these three families showed a common, disease-associated haplotype. Our findings further support the notion that this mutation has probably common ancestral origins in this area. Similarly, c.1408-2A > G (IVS10-2A > G) in *PDE6A* was reported in Pakistani individuals with recessive RP^23^. The relatively high prevalence of these mutations indicates once more an ancestral origin and provides a rather strong element for performing targeted molecular diagnosis of IRDs in this region.

The *CNGA3* mutation c.1306C > T (p.R436W) identified in family MA25 is relatively frequent in achromatopsia cases^33^. This variation has mostly been recorded in a compound heterozygous state with other mutations^25^ and was initially thought to be a variant that was limited to German patients^33^. Subsequent studies have shown that this mutation is present in other populations^41^, as we also report in this work, and therefore represent a common cause of achromatopsia worldwide.

In conclusion, our study confirms the power of homozygosity mapping for identifying pathogenic variants in consanguineous families with IRDs. This approach is precious to provide correct clinical diagnosis and genetic counseling in isolated areas of Pakistan, raising at the same time awareness about IRDs and the risks of intermarriage.

## Author Contributions

M.A.N.S., K.N., K.M.N., M.A., and C.R. wrote the manuscript; M.A.N.S., K.N., G.V., M.A., and C.R. designed the study; M.A.N.S., K.N., E.U., F.S.K., J.I., R.B., A.J., S.S., and G.V. performed data acquisition. All authors analyzed the data and reviewed the manuscript.

## Additional Information

**How to cite this article**: Saqib, M. A. N. *et al.* Homozygosity mapping reveals novel and known mutations in Pakistani families with inherited retinal dystrophies. *Sci. Rep.* **5**, 09965; doi: 10.1038/srep09965 (2015).

## Supplementary Material

Supporting Information Supplementary Tables S1-S3

## Figures and Tables

**Table 1:** Clinical features of the patients examined.

**Family**	**MA62**	**MA94**	**MA132**	**MA123**	**MA25**	**MA69**	**MA117**	**MA97**
Individual	V−1	V−3	IV−3	IV−4	VI−4	IV−12	IV−2	IV−1
Age (Years)	NA	NA	18	23	NA	15	47	24
Age of onset	1st decade	Infancy	1st decade	2nd decade	Infancy	Infancy	2nd decade	1st decade
Legally blind	+	−	−	−	−	−	−	+
Photophobia	−	++	−	+++	++	+	+	−
Nystagmus	−	+	−	+++	++	+	++	−
Night blindness	+	−	+	−	−	−	−	+
Visual Acuity	LP	20/60	20/40	NA	20/40	20/200	20/80	NLP
ERG (Rod)	NA	Normal	Reduced	NA	NA	Normal	Reduced and delayed	Absent
ERG (Cone)	NA	Reduced	Normal	NA	NA	Reduced	Delayed	Low

+ and − symbols indicate presence/absence, as well as degree of a given feature (+ mild, ++ moderate, +++ severe). NA, not available; LP, light perception; NLP, no light perception.

**Table 2:** Mutations identified in this study.

**Family**	**Gene**	**RefSeq ID**	**Nucleotide variant**	**Protein variant**	**Polyphen**	**SIFT**	**Mutation Taster**	**Previously reported**
MA62	*RHO*	NM_000539	c.448G > A	p.E150K	Probably Damaging	Deleterious	Disease causing	Yes^21^
MA94	*CNGB3*	NM_019098	c.646C > T	p.R216X	Probably Damaging	Deleterious	Disease causing	Yes^22^
MA132	*PDE6A*	NM_000440	c.1408-2 A > G	Splice defect	NA	NA	NA	Yes^23^
MA25	*CNGA3*	NM_001298	c.1306C > T	p.R436W	Probably Damaging	Deleterious	Disease causing	Yes^25^,^26^
MA69	*CNGA3*	NM_001298	c.991G > C	p.G331R	Probably Damaging	Deleterious	Disease causing	No
MA117	*RPGRIP1*	NM_020366	c.2656C > T	p.L886F	Damaging	Deleterious	Disease causing	No
MA97	*CNGB1*	NM_001297	c.413-1G > A	Splice defect	NA	NA	NA	No
