# Supplementary material for: Homozygosity mapping reveals novel and known mutations in Pakistani families with inherited retinal dystrophies
Source: Sci Rep. 2015 May 6;5:9965. doi: 10.1038/srep09965 (PMC4421863; doi:10.1038/srep09965)
Supplement: Supporting Information — Supplementary Tables S1-S3 [file srep09965-s1.pdf]

SUPPLEMENTARY INFORMATION

Homozygosity mapping reveals novel and known mutations in Pakistani families with inherited retinal dystrophies

Muhammad Arif Nadeem Saqib, Konstantinos Nikopoulos, Ehsan Ullah, Falak Sher Khan, Jamila Iqbal, Rabia Bibi, Afeefa Jarral, Sundus Sajid, Koji M. Nishiguchi, Giulia Venturini, Muhammad Ansar, Carlo Rivolta

Supplementary Table S1. Primers used in this study.

Exon	Primer	Product size (bp)
CNGB1Ex2F	GCTGATCTCAAACTCCTGGA	
CNGB1Ex2R	GCCAACCTTTTCTGCTGTAG	478
CNGB1Ex3F	CTTGAGGGAGAATCAGGCTG	
CNGB1Ex3R	CCACCCTTAGCTTCTCTGAG	273
CNGB1Ex4F	CTCTCCCCTGACTTGATCTC	
CNGB1Ex4R	TCAAGGGGCTAACAGTCTAA	492
CNGB1Ex5-6F	AGGGACAGAGACTCACCTAG	
CNGB1Ex5-6R	CAGTCAGGTGAGAAGATCCA	430
CNGB1Ex7F	TGACCCTGCTTTGAACCTG	
CNGB1Ex7R	AAACAGGCCCAGAAGAGAC	300
CNGB1Ex8F	TGAGAGTTTCCAGGACAAGG	
CNGB1Ex8R	AGTCACTCCTCCCCATAGAG	379
CNGB1Ex9F	CGGGATTCAGAAGGAGAGAC	
CNGB1Ex9R	TTACCCAAGACCCACAAGT	413
CNGB1Ex10F	GGTCTGTTTGAGGATTGGGA	
CNGB1Ex10R	GAAAATGTGGGAAAGGGCAC	492
CNGB1Ex11F	TTGTTTTCTGCCTTGGGAAC	
CNGB1Ex11R	AGGGAGTGGGTGGAATGAA	319
CNGB1Ex12F	AGGGACTCTCTTCTCCATCC	
CNGB1Ex12R	CTACCTGGCTACCTGAACAG	375
CNGB1Ex13F	ACTGCATGACTAAGCACTGA	
CNGB1Ex13R	GGGAGAAGGATGGCTGTATG	429
CNGB1Ex14F	CCTACAGAAAATAGAGGCAGGA	
CNGB1Ex14R	CAGCCTTACACAGCACAGAA	292
CNGB1Ex15F	TTCTTGGGCATCTCTGTACC	
CNGB1Ex15R	CCCTGTAAGCAAAGTGGACA	295
CNGB1Ex16F	AAGGCAGATCTAACAGGAGC	
CNGB1Ex16R	AAGGAGATGGAGGGAGAGGA	354
CNGB1Ex17F	CCCTCCAAACATGCTTTCTC	
CNGB1Ex17R	CCAACTCGCTGTGTGATTTT	385
CNGB1Ex18F	TGGCTCTAGATGATCTTTAGGG	
CNGB1Ex18R	CTCAGCCTCTCCTTCACAAG	491
CNGB1Ex19F	TCCCGGACTGAATTTCCTTC	
CNGB1Ex19R	GATCCCAAATCCAGAGAGTG	496
CNGB1Ex20F	TCTCTCTGCATCCTCTACCT	
CNGB1Ex20R	CAGGCAAGAAACTGTGGAAT	388
CNGB1Ex21F	AGTGCCACATGTAGTAGTTGA	
CNGB1Ex21R	TATTGAACAAGCACAGCAGC	465
CNGB1Ex22F	GATGGTCCTTGGGCTGTTAT	
CNGB1Ex22R	CTTGTGGTTTGGAGGACACT	323
CNGB1Ex23F	GAGGATAATGAAGCTGCCCT	
CNGB1Ex23R	ATAAATGCATCTCCCCTCGG	439
CNGB1Ex24F	GGGCTGAGGACTTCTGATAT	
CNGB1Ex24R	TGTTGAATGTTGTGTGTGCA	377
CNGB1Ex25F	GGTGCAATCTCAACAAAGCA	

CNGB1Ex25R	CCTTGTCATGCCAGTGATTG	393
CNGB1Ex26F	GGGTTTCTGTCTTTTGCACA	
CNGB1Ex26R	GGTCTCTGCAGAAAACCATT	521
CNGB1Ex27F	CCCGTCAGTAAAACTTTCTCTG	
CNGB1Ex27R	TCAGAGACACAGAGGACGA	436
CNGB1Ex28-29F	TGCACTACAAATCCAGAGTG	
CNGB1Ex28-29R	CAGCTCAGTTCCTTGAAAGC	483
CNGB1Ex30F	AGTCTACAGATGGGGTGACT	
CNGB1Ex30R	CCCACACAGATGAGAACCTT	472
CNGB1Ex31F	ACCACTGTCCTCCACACAT	
CNGB1Ex31R	ATGATCACTCCACTGCACTC	384
CNGB1Ex32F	AATCACCACCACTACACAGC	
CNGB1Ex32R	CTTGCACTCTGTCACTGGTA	484
CNGB1Ex33F	CACCCCACCCACTAGATTTC	
CNGB1Ex33R	AAGCATCTTCTCTTGAGCCG	549
CNGB3Ex1F	AGCTAAGGAGTTGCCTGTAGGA	
CNGB3Ex1R	CAGATGGTGCCAGGTAAAACTA	397
CNGB3 Ex2F	TGTGGAGGCTGAGGTTGATT	
CNGB3 Ex2R	TCATCAGACAGCACATTTCA	357
CNGB3Ex3F	GCCTGAAGGTGTCTACCAGT	
CNGB3Ex3R	AGCTAAAGGGGAGAGTGGAT	494
CNGB3 Ex4F	ATTCCACCAGCACTATTTCC	
CNGB3 Ex4R	CCTCAGCACTTCTTTCTTCC	372
CNGB3 Ex5F	GCGGTGTTTGGTTAAGAAAT	
CNGB3 Ex5R	CAGGGTTTCTTGGTGATAGC	294
CNGB3Ex6F	GTCCAGAGGCAGAATGGTGT	
CNGB3Ex6R	TTCTTGCAATTATCCATGCAG	484
CNGB3 Ex7F	GAACCAACCAAGAGAAACAGTTA	
CNGB3 Ex7R	AAAGTCAAAATGGTAATAGATCACG	200
CNGB3 Ex8F	TGCAACGCCTAAGGAGAGAT	
CNGB3 Ex8R	AGCATTGATGAGGGCAGAAA	491
CNGB3Ex9F	AAAATGTACTGTCCAGAGGAAAA	
CNGB3Ex9R	GGGTCATATCCCTGCCAAAT	316
CNGB3 Ex10F	TGGCACCATCACTTTGTACTG	
CNGB3 Ex10R	AGCATTTACCAGCCATTGAATG	399
CNGB3Ex11F	TTCTCCCAAGAATAGTGGTCTTT	
CNGB3Ex11R	CATCCTCCTTCAACTCATTAAAA	362
CNGB3Ex12F	TGAAAAGTTGTCTGGGCAAG	
CNGB3Ex12R	TTCAACCTTTTGTTCAAATCCA	426
CNGB3Ex13F	TGTTTGCATTTTGTTTCCTTT	
CNGB3Ex13R	GGTTCTAAAGAATAAGCCGTTGG	398
CNGB3 Ex14F	CCATAGCCATTGGCAGTTA	
CNGB3 Ex14R	ATGTCCGAAATCCTCAAATG	280
CNGB3 Ex15F	TGTTCAACCCATGTCTGTAAA	
CNGB3 Ex15R	AAATCTGAGCGGGAACTTATT	382
CNGB3 Ex16F	GCCATTTCTATTCCCACATC	
CNGB3 Ex16R	AAACCAACTCCATCCATCTC	362
CNGB3 Ex17F	ACTTGATCACAGTGAGATATGCT	

CNGB3 Ex17R	GTCCAAATCATCCCAGTGTCTG	434
CNGB3Ex18F	GCCATTGCATGCTAATCAAA	
CNGB3Ex18R	TGCATGAAATCACACTCTCAGAT	573
PDE6AEx1F	CTTAATCTCCCAAGCTTGCTC	
PDE6AEx1R	TTGTCACCAGCCTTGTCTTG	742
PDE6AEx2F	GCCCCTGTTGAAGAGGAATA	
PDE6AEx2R	GAAAACTGAGGCCAGGTCAA	370
PDE6AEx3F	TTGTCTCTGATGAAGCTTTGAA	
PDE6AEx3R	TAGGCACCTTCATTCCCATC	297
PDE6AEx4F	TGGATTATTGTGAAGGGTAAATG	
PDE6AEx4R	GCCAAGACTCTAGCCGTCAG	340
PDE6AEx5F	TGTTAGCTGACTCATGGAGGTG	
PDE6AEx5R	TCCTTTAACAGGGTTTACTGTGC	237
PDE6AEx6F	AGATCAAGCCATTGCACTCC	
PDE6AEx6R	TGCTTGTCTTTCTTCAGTCCTTC	325
PDE6AEx7F	CCAACATTTTGGGTTTGGTC	
PDE6AEx7R	TCCTTCCACTCTTTCTTCCA	276
PDE6AEx8F	GACAAGAACATGGTGTTTCTGA	
PDE6AEx8R	CCCCATTTCCATTGGTACAG	300
PDE6AEx9F	TCTCAGCCAATCAGGGTCAT	
PDE6AEx9R	GCAGTGAGAGTGAAGAAAGTGG	396
PDE6AEx10F	TCAAACCCATTTTATAGCTGAGA	
PDE6AEx10R	GCTAGGCAGGAAGGAACACA	400
PDE6AEx11-12F	GAGCTGGTGGTTCTGTTGC	
PDE6AEx11-12R	GCAAGCAAGAAAGAAAATTAAAA	587
PDE6AEx17F	GCCTTGGCTCTTTCAACCTT	
PDE6AEx17R	TGAAGGCCTCCAAAATGAAA	362
PDE6AEx13F	TCACCTCCTGGTCCAATGAT	
PDE6AEx13R	AGCCCGTACTGCTTTCACAT	397
PDE6AEx14F	TTTTAGTTCTGGGCCCTCCT	
PDE6AEx14R	AGACTTCCCTGTTGGCCAGT	300
PDE6AEx15-16F	TCACTTGTGGAGAAGGCTGA	
PDE6AEx15-16R	CCTGGGCAACAGAGTGAGAT	383
PDE6AEx18-19F	GATGCAATGGGGGTGAGC	
PDE6AEx18-19R	CTGCATTAGGAAAAGGTCATCT	700
PDE6AEx20F	AAGGAGCATACTGCCTCTGG	
PDE6AEx20R	GTTGTCTGGAAATCCCAGGT	487
PDE6AEx21F	GCTACTCCGAAGCAGCTCAT	
PDE6AEx21R	GCCTGAATGAGACTCCGTGT	361
PDE6AEx22F	TTTGCCTTCTGCATGTGTTG	
PDE6AEx22R	CAGGGAAGCCAAAAGATTGA	376
RHOEx1F	TGCAGCGGGGATTAATATGA	
RHOEx1R	ATTGACAGGACAGGAGAAGG	684
RHOEx2F	CCTGGAATTTCTTTGCCCAG	
RHOEx2R	GATTCTGTTTGACATGGGGC	599
RHOEx3F	GAATGTGAAGCCCCAGAAAG	
RHOEx3R	CTGGTGGGTGAAGATGTAGA	518
RHOEx4F	TATGGGCAGCTCGTCTTCA	

RHOEx4R	CTGCATTTCTCACACACTCC	542
RHOEx5F	CAGTTCCAAGCACACTGT	
RHOEx5R	AAGGAGCCTATGTGACTTCG	290
KCNV2Ex1-1F	CGAGGTTGACCGACATTTTT	
KCNV2Ex1-1R	GCACCCAGGGAGCAAATG	506
KCNV2Ex1_2-F	TCCTCCTAGAGGCAGTGAGC	
KCNV2Ex1_2-R	GTAGACCAGCTGGAAGACGG	565
KCNV2Ex1_3-F	CTAAGCCTGTGCGACGACTA	
KCNV2Ex1_3-R	GCAGGTACTCGAGCGTGAAG	550
KCNV2Ex1_4-F	TTCTTCACGCTCGAGTACCTG	
KCNV2Ex1_4-R	TTTTGTTAGCTGTATGCGGCG	567
KCNVEx1-3-4 R	CTTGAGGATGCGGAAGATG	
KCNV2Ex2_1-F	TTCTTCTCCTCCCCGATCTT	
KCNV2Ex2_1-R	TCTAAGCTGTTGCTCCCCTC	597
KCNV2Ex2_2-F	TGGCCCAAACTCAGAATGTC	
KCNV2Ex2_2-R	CTCCCTCCCTCTGGAAAGTT	223
CNGA3Ex1F	TGTGTGTGTCCCCACCTTTA	
CNGA3Ex1R	CCAGCTCAAGAGCCCCTAA	453
CNGA3Ex2F	GATGAGCTGGGTTTGCAGTT	
CNGA3Ex2R	GACCAGAGCCAGGCATAAAA	250
CNGA3Ex3F	GGGCTTGAAATCAATTCTGC	
CNGA3Ex3R	CCCCATCTAGCACTTTTTCC	238
CNGA3Ex4F	TTCCTCTCCCTCTGGCTCA	
CNGA3Ex4R	AACAGGATGGAGCAAAGCAC	298
CNGA3Ex5F	CCCAAGGAATGGAAACAGAG	
CNGA3Ex5R	TAAGGAGAGAGGCCAAGCTG	193
CNGA3Ex6F	CAAAGCTACAGTCTTGGAGCA	
CNGA3Ex6R	CCTGGTTTCCCCCTTTCC	216
CNGA3Ex7F	CTCCAGAAACACACGCACAG	
CNGA3Ex7R	TAATGTCCCATCCACCATGC	238
CNGA3Ex8aF	AAAAGTCAGCCTCTGTGATGC	
CNGA3Ex8aR	AGGCTCTTGAGCACCTCCTT	753
CNGA3Ex8bF	CCAACAAGAAGACGGTGGAT	
CNGA3Ex8bR1	TGCTGCATTTTCACTGTTGTT	759
CNGA3Ex8bR2	AAAAAGCCTAAAAACACCTTGC	1090
RPGRIP1Ex1F	TGTCCACACTACCATGAGAATGA	
RPGRIP1Ex1R	CTGGGTGACAGAGCGAGACT	345
RPGRIP1Ex2F	TGCTCTCTGGACAAGATGTGA	
RPGRIP1Ex2R	GGCAGGAGAATTGCTTGAAT	372
RPGRIP1Ex3F	TGTACTGGGGACAGAAGGCTA	
RPGRIP1Ex3R	AAACGTGGCTGGCACATC	498
RPGRIP1Ex4F	AAGGGTTTCACCCAGGTCTC	
RPGRIP1Ex4R	AAAATTAGCTGGGCATGGTG	669
RPGRIP1Ex5F	TCCTCGACATGTACCAAGGTT	
RPGRIP1Ex5R	CCTCTGAGATGGAGGAAAGG	410
RPGRIP1Ex6F	GTAAGACGGGAAGGCAAGAG	
RPGRIP1Ex6R	TTGTGAGGCTTGGATTTGAA	386
RPGRIP1Ex7F	TGATTTGTTTTGTGGGATTTTT	

RPGRIP1Ex7R	AATTTGCTCCAGCAATAGGC	409
RPGRIP1Ex8F	TCCAGAGAAATGCTAGGGTGA	
RPGRIP1Ex8R	ACTGCTAGGGAAGCAGCAAA	554
RPGRIP1Ex9F	AGTTGGATGGCTTCTCAGAC	
RPGRIP1Ex9R	AGTGTCAAATTCCCTTCCAAAAG	370
RPGRIP1Ex10F	TGGGAAATGGATATGACTTGG	
RPGRIP1Ex10R	CCCAAACCAGAGGTAGAGGA	472
RPGRIP1Ex11F	CACTTGATCCAACTCTGACCA	
RPGRIP1Ex11R	CAGCGAGACTCTGTCTCAAAAA	382
RPGRIP1Ex12F	ACCAACAACTGTTTTATGGAGAA	
RPGRIP1Ex12R	GGCATGTGTGAGTCCTCAAG	398
RPGRIP1Ex13F	GGGTCTGCAAGGAAATCAAA	
RPGRIP1Ex13R	ATGAGAGGCACCCTTCTTGA	396
RPGRIP1Ex14F	TGGACTTCCACCATGTGTTT	
RPGRIP1Ex14R	TACAGATGGTGTGGCAAGGA	685
RPGRIP1Ex15F	TGTTCTTGATCCTTGCCACA	
RPGRIP1Ex15R	CTCATGAGCTGTTTGGCTGA	271
RPGRIP1Ex16F	GTTTGCAGGCAGGTGAAGAT	
RPGRIP1Ex16R	TCTGCTCTGTTGCTCTTGACA	649
RPGRIP1Ex17F	TGCACTGCTGTTTGATTTTACTT	
RPGRIP1Ex17R	TGAGCCACTGCACCTACTTC	464
RPGRIP1Ex18F	TCAGCTCCATGAGGAGAGAAA	
RPGRIP1Ex18R	CCCAAAGTGCTGGGATTACA	472
RPGRIP1Ex19F	GCCTGAGTGACAGAGGGAGA	
RPGRIP1Ex19R	GCTGCTCTTGAAAGCCTGAT	385
RPGRIP1Ex20F	GATGTGTGTGCTGGGTCTTTT	
RPGRIP1Ex20R	CATGACTGGCCACTGTCATTA	242
RPGRIP1Ex21F	TGCCAGTTTTACCTATAAAAGGA	
RPGRIP1Ex21R	TGTTCATCAGACTTCCTCACC	400
RPGRIP1Ex22F	AGACTGCGGTGAACCAAGAT	
RPGRIP1Ex22R	GCATCAGCACAAAACCAAAC	400
RPGRIP1Ex23F	AAGTGATTCAGAGAAGACGTTGT	
RPGRIP1Ex23R	TGTAGGGATAAGATTTCAATCCA	373
RPGRIP1Ex24F	CCCAGTACCTAACCTGACAAAT	
RPGRIP1Ex24R	GCCTGGTAAAGTGCTAAGGTACT	400

Supplementary Table S2. Individuals genotyped for homozygosity mapping

Family ID	Genotyped individuals
MA97	III-1, III-2, IV-1, IV-2, IV-5, IV-6
MA62	IV-1, IV-3, V-3, V-4, V-5, V-6, V-7, V-8
MA132	III-1, III-2, IV-3, IV-4, IV-5, IV-1
MA25	IV-1, IV-2, V-1, V-3, V-4,VI-1, VI-2, VI-4, VI-5
MA69	III-3, III-4, IV-10, IV-11, IV-12, IV13, IV-14
MA94	IV-1, IV-2, V-3, V-4, V-5
MA117	III-2, IV-1, IV-2, IV-3, IV-4
MA123	III-1, IV-2, IV-3, IV-4

Supplementary Table S3. Homozygous regions identified

Family ID	Homozygous Region			Known IRD gene (if any)
	Chr	Flanking SNPs or genomic coordinates	Region (Mb)	
MA69	2	rs17041053-rs17652317	18.7	*CNGA3*
	6	rs6596790-rs1887507	6.7	-
	8	rs7006666-rs2705017	3.9	-
MA94	7	rs727531-rs764891	36.6	*IMPDH1*
	8	rs4629903-rs28624756	50.5	*CNGB3*
MA25	2	g.85,488,120-105,479,784/hg19	19.9	*CNGA3*
MA97	8	rs11984569-rs12542396	29.40	-
	9	rs12344716-rs12236186	3.31	-
	16	rs12596791-rs11866632	47.25	*CNGB1*
MA132	5	rs3995091-rs7724036	2.43	*PDE6A*
MA62		rs11923216-rs17301766	6.43	*RHO*
MA117	4	rs6534397-rs11947641	2.41	-
	8	rs4873772-rs11947641	2.40	-
	10	rs7093966-rs11592507	3.14	-
	14	rs7148898-rs12892350	4.96	*RPGRIP1*
MA123	2	rs13384785-rs6708727	11.64	-
	9	rs7860758rs10756524	11.85	*KCNV2*
